# Supplementary figures and images for: IQCELL: A platform for predicting the effect of gene perturbations on developmental trajectories using single-cell RNA-seq data
Source: PLoS Comput Biol. 2022 Feb 25;18(2):e1009907. doi: 10.1371/journal.pcbi.1009907 (PMC8906617; doi:10.1371/journal.pcbi.1009907)

Fig. S1

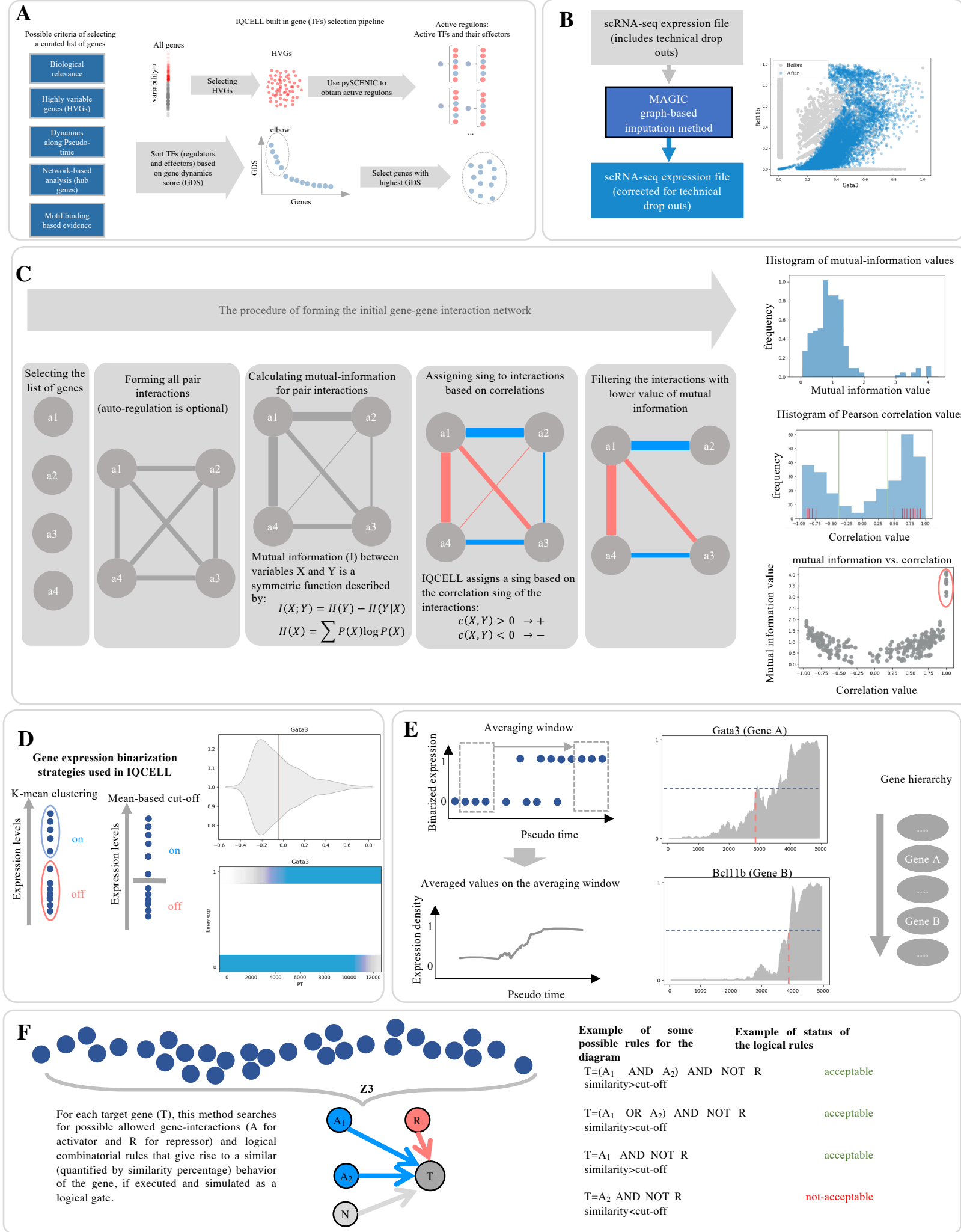

Supplement: S1 Fig — (A) Overview of gene selection criteria (left). IQCELL’s gene selection pipeline (right). After selecting HVGs, IQCELL uses pySCENIC to select active regulons (TFs and their effectors), and finally IQCELL uses GDS to rank and select TFs for the final list. (B) Overview of gene expression recovery step. The scRNA-seq data is corrected for dropout effect via a borrowed library ‘MAGIC’ from literature (left). Raw (grey) and recovered (blue) expression of Bcl11b vs Gata3 (right). (C) Overview of generating the initial interaction network. The steps toward obtaining the directional and signed interaction network from an initial list of genes (left column). The histogram of mutual information between gene pairs, the histogram of Pearson correlation between gene pairs (the correlation value of 1 is for the correlation of genes with themselves, marked by red), and mutual information vs. correlation values (right column). In the histogram of Pearson correlation values, the interactions that end up in the final model are marked by short red lines and the vertical green line shows the sign cutoff (which its value has been chosen to be permissive); results are for the T-cell dataset. (D) Overview of expression binarization step. There are two implemented binarization methods in IQCELL. K-means clustering (default) and binarization based on the mean value of expression of the gene between all the cells (left). Example of binarization of genes and their expression along the pseudo time (right). (E) Overview of generating the gene hierarchy step from the binarized gene expressions. First, the expression levels are averaged with a sliding window along the pseudo-time. This results in the density profile of binarized genes along the pseudo-time (left). Next, based on clustering the density, the transition point (from high to low or low to high) are captured (center). Finally, genes are sorted based on transition points. Genes can interact with genes with a lower rank (right). (F) Ov [file pcbi.1009907.s001.pdf]

Fig. S2

A

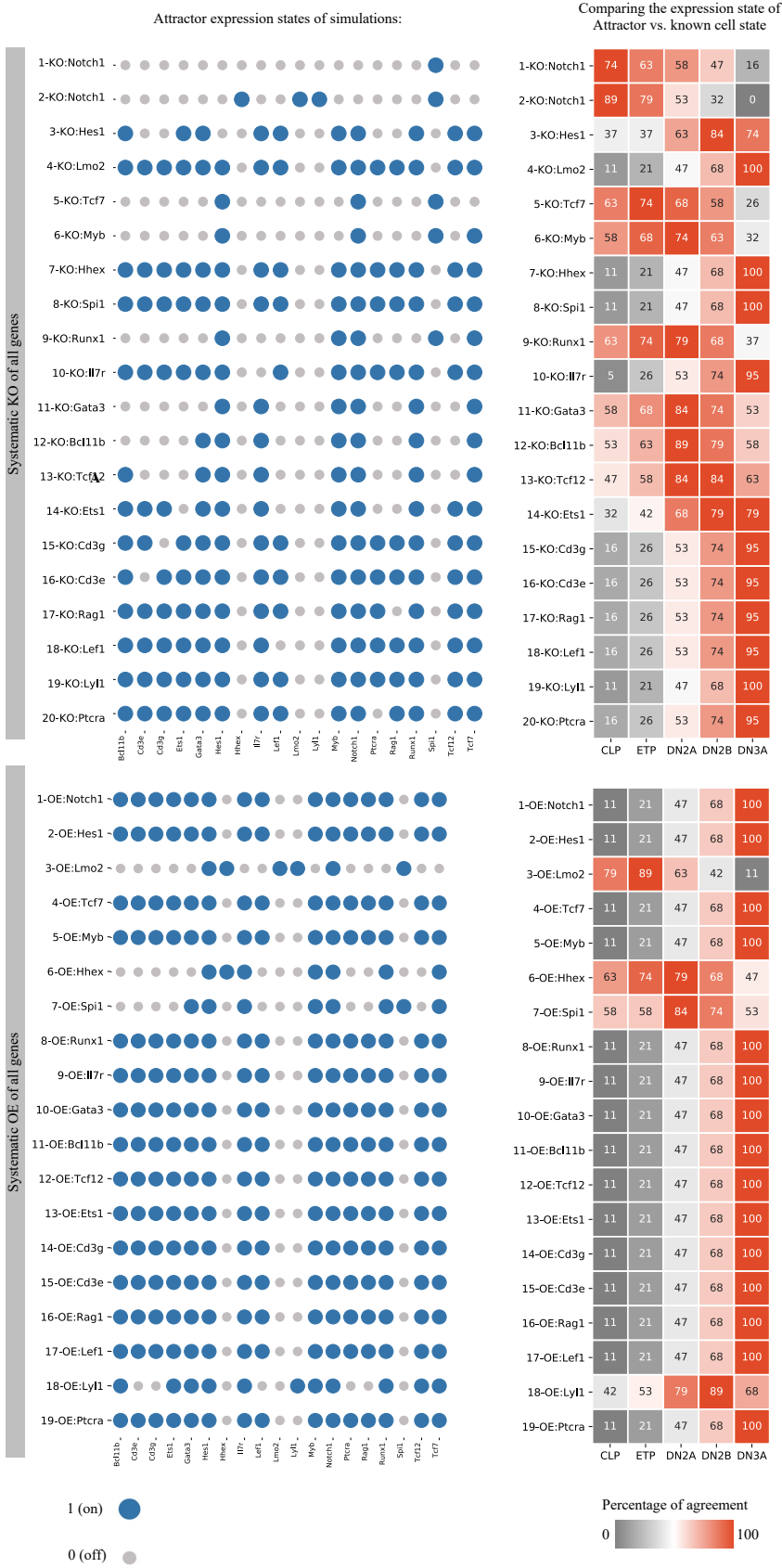

B

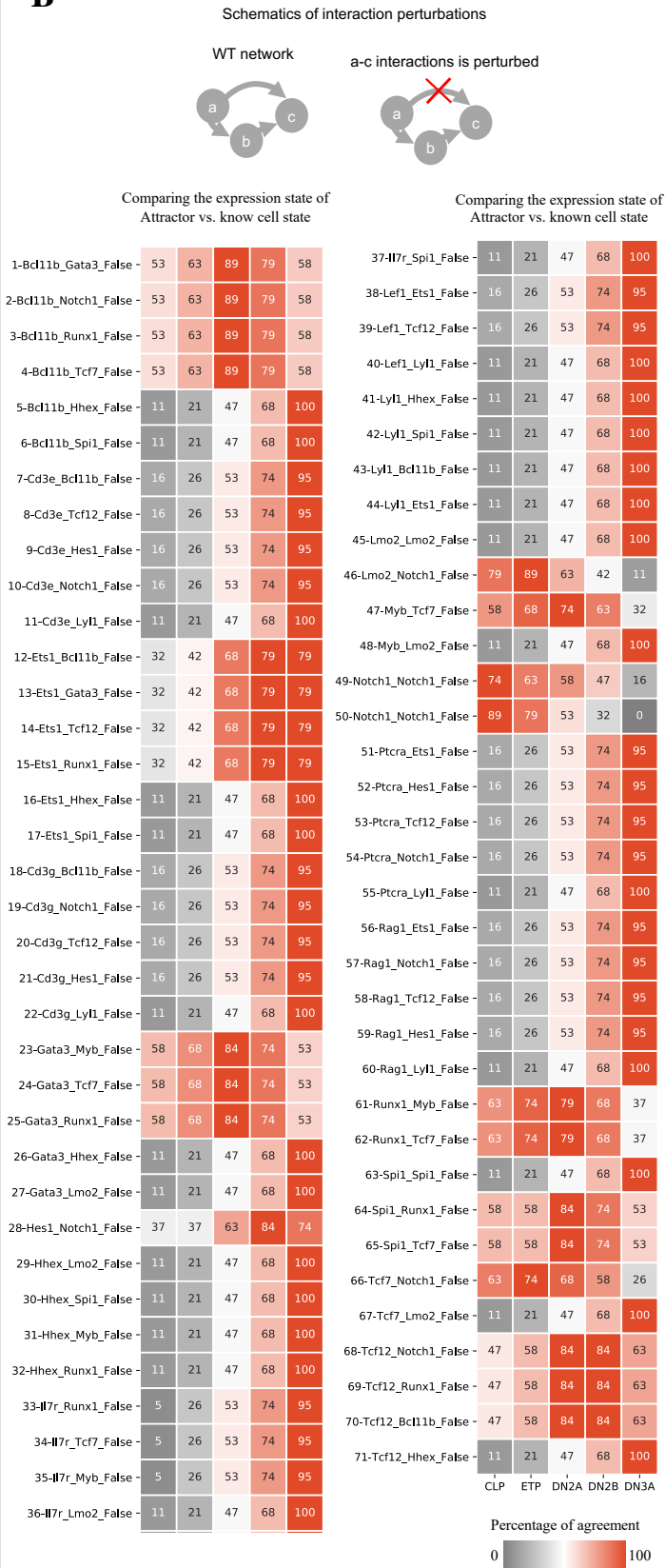

Supplement: S2 Fig — (A) Systematic gene perturbations. The expression states of the model attractor (left). The percentage of similarity between the attractors (vertical axis) and the binarized expressions of CLP, ETP, DN2A, DN2B, and DN3A cells (horizontal axis) [38] (right) for systematically perturbed GRN with single gene KO and OE. (B) Systematic gene-gene interaction perturbations. Overview of GRN link perturbation (top). The percentage of similarity between the attractors (vertical axis) and the binarized expressions of CLP, ETP, DN2A, DN2B, and DN3A cells (horizontal axis) [38] (bottom) for systematically perturbed GRNs. (PDF) [file pcbi.1009907.s002.pdf]

Fig. S3

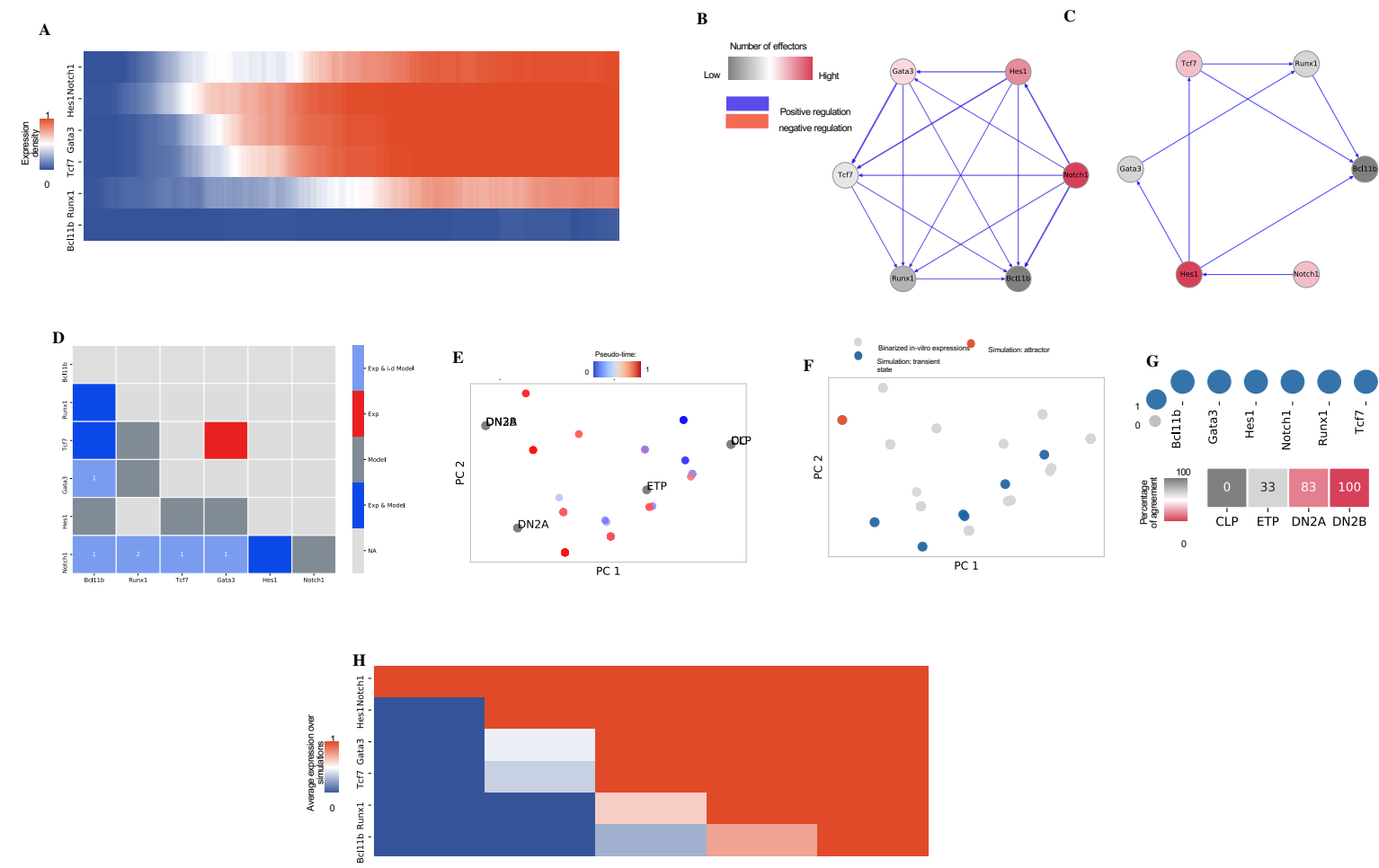

Supplement: S3 Fig — (A) To demonstrate the universality of IQCELL, we have tested this platform with another in-house scRNA-seq data. The IQCELL tutorial in the IQCELL website is based on this dataset. We used 10X scRNAseq by performing whole-genome transcriptional analysis. These experiments are performed with the mouse T-cell progenitor populations from fetal liver (FL) hematopoietic stem and progenitor cells (HSPCs) differentiated in vitro using the DL4+VCAM platform [81]. FL HSPCs were seeded on DL4+VCAM coated plates and cultured for 4 or 7 days prior to analysis, or immediately sorted and captured for library preparation. Pooling cells from multiple differentiation time points enabled sampling of cells from the entire T-cell lineage progression, rather than just endpoint transcriptional states. Here we have selected a small set of 6 genes that are important in the early T-cell development (from ETP to DN2 stages). The heat map shows the expression matrix of smoothed binarized expressions along the pseudo-time. (B) The set of all possible gene-gene interactions, filtered by interaction hierarchy and mutual information cut-off (the thickness of lines represents the mutual information between genes), and signed by correlation. (C) Provisional GRN for early mouse T-cell development. (D) Detailed representation of the proposed interactions provided by IQCELL and experimentally reported ones. (E) PCA plot of the binarized scRNA-seq data color coded with the pseudo-time values attributed to each cell. The binarization is performed by clustering the scRNA-seq expressions into expressed or not expressed levels. On top of that, the binarized expressions of CLP, ETP, DN2A, DN2B, cells have been calculated from the Immgen microarray data [38] and overlaid on RNA-seq data. (F) PCA plot of the simulated developmental trajectories are overlaid on the binarized scRNA-seq. (G) Expression states of the model attractors (top). The percentage of similarity between attractor and known cell states [38 [file pcbi.1009907.s003.pdf]

Fig. S4

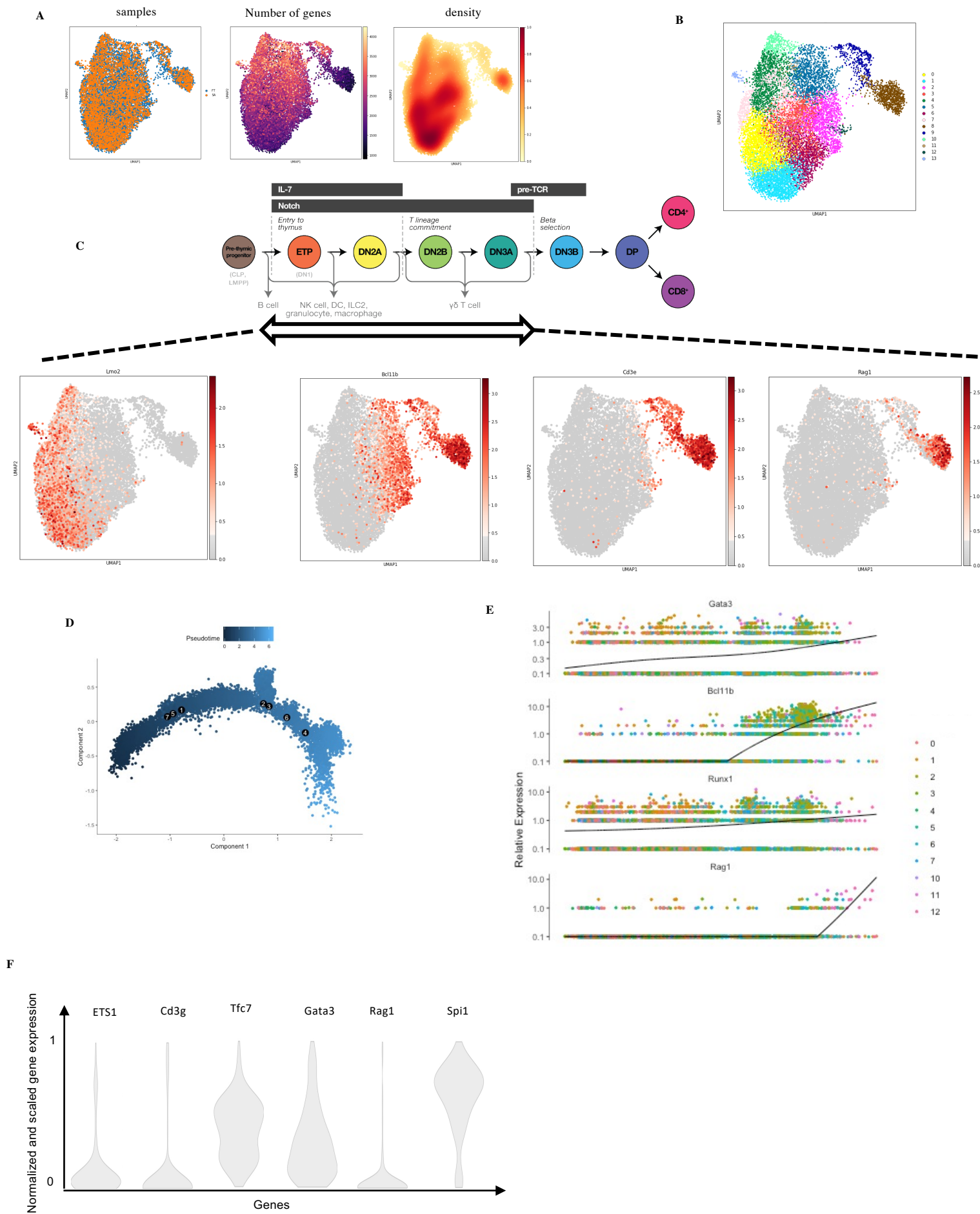

Supplement: S4 Fig — (A) UMAP representation of data overlaid with sample ID, number of genes per cell, and density plot. (B) Clustering of scRNA-seq data. (C) Gene expression of stage-specific gene overlaid on top of UMAP. (D) The pseudo-time trajectory of the data inferred by Monocle platform. (E) Example expression of genes along the pseudo-time trajectory. (F) The normalized and scaled to 1 expression of some example genes. (PDF) [file pcbi.1009907.s004.pdf]

Fig. S5

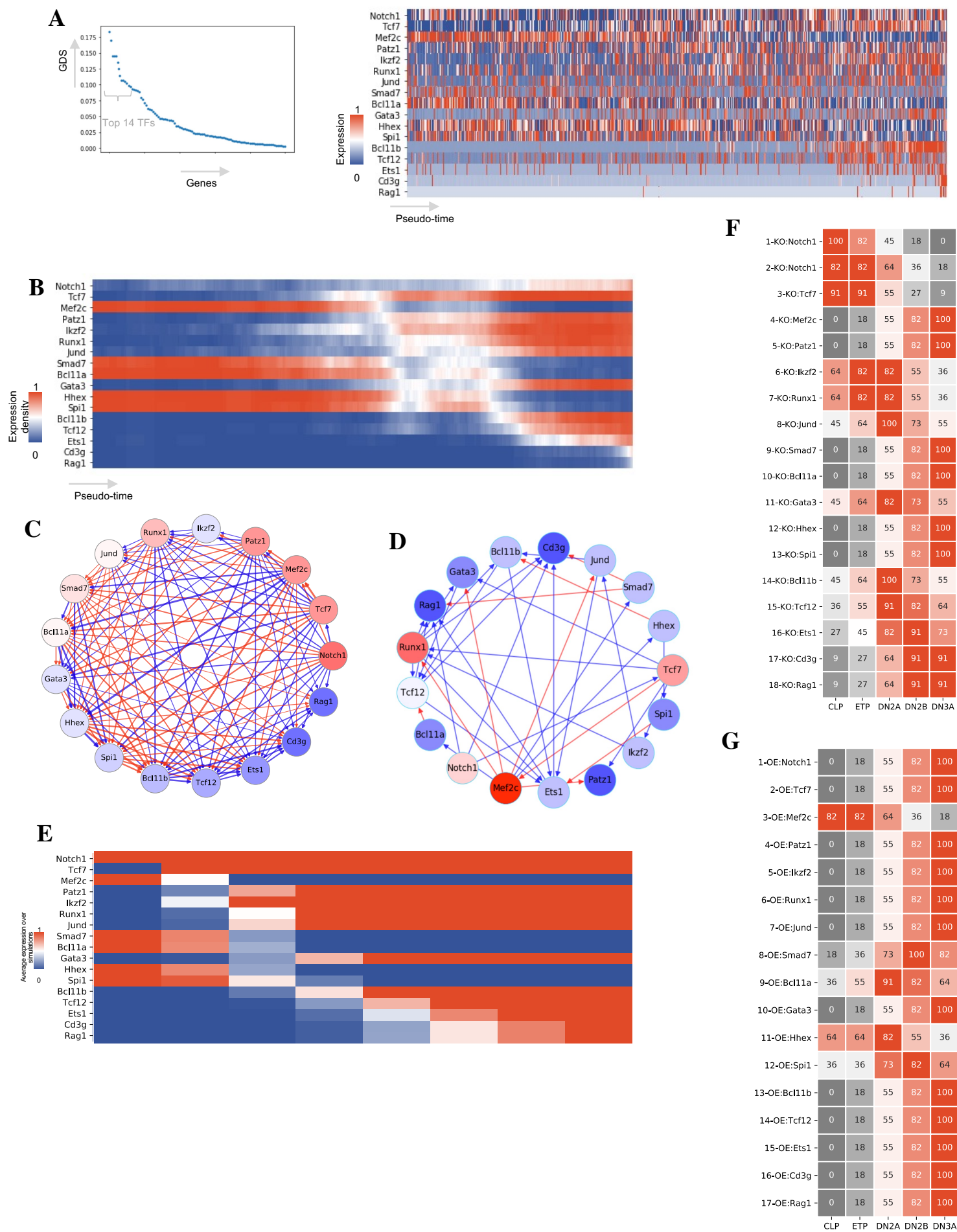

Supplement: S5 Fig — (A) The GDS of genes, the top 14 genes have been selected (left). Log transformed expression matrix for selected genes from sc-RNA data along the pseudo-time axis. Gene expression is corrected for dropout effects using MAGIC. Red indicates high expression, blue indicates low expression (right). (B) Smoothed binarized gene expression matrix (expression density). Gene expression values were binarized by clustering, averaged over a pseudo-time window, then sorted based on transition points from early to late. Red indicates high expression, blue indicates low expression. (C) The set of all possible gene-gene interactions, filtered by interaction hierarchy and mutual information. (D) The provisional GRN for early mouse T-cell development. (E) Average gene expression at each simulation step. All simulations started from the same initial condition (ETP) and move toward the same attractor (*). (F) Systematic KO: The percentage of similarity between the attractors (vertical axis) and the binarized expressions of CLP, ETP, DN2A, DN2B, and DN3A cells (horizontal axis). (G) Systematic OE: The percentage of similarity between the attractors (vertical axis) and the binarized expressions of CLP, ETP, DN2A, DN2B, and DN3A cells (horizontal axis) (PDF) [file pcbi.1009907.s005.pdf]

Fig. S6

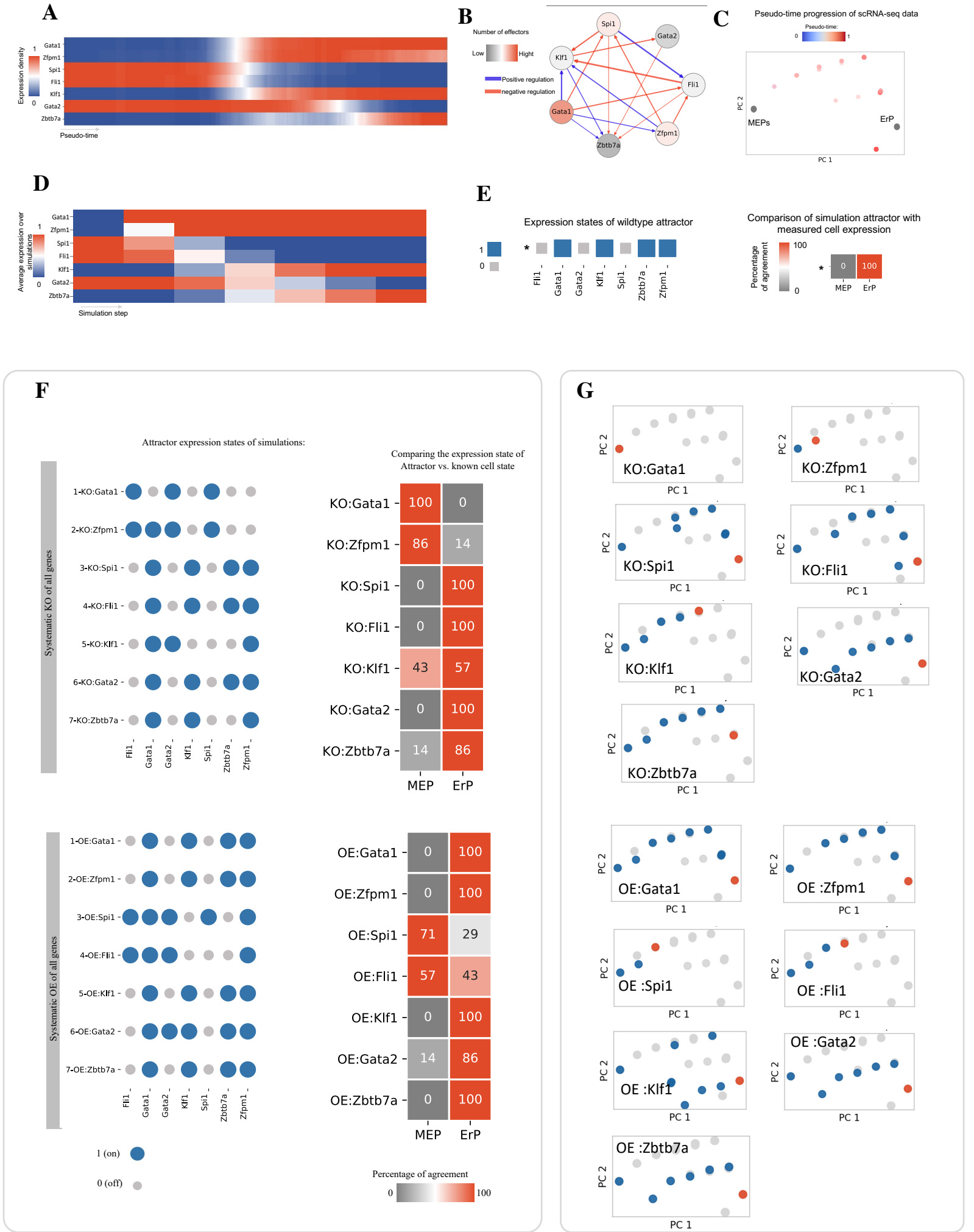

Supplement: S6 Fig — (A) Average gene expression at each simulation step. All simulations started from the same initial condition (MEPs) and move toward the same attractor (*). (B) The set of all possible gene-gene interactions, filtered by interaction hierarchy and mutual information (and signed by correlation. Positive and negative interactions are represented by blue and red edges, respectively. Edge width represents the relative amount of mutual information of the interaction. Nodes colored red have higher out-degrees. (C) The PCA plot of the binarized scRNA-seq data color coded with the pseudo-time values attributed to each cell. The binarization is performed by clustering the scRNA-seq expressions into expressed or not expressed levels. (D) Average gene expression at each simulation step. All simulations started from the same initial condition (MEPs) and move toward the same attractor (*). (E) Expression states of the GRN model steady state attractors. Genes that are expressed (1) and not expressed (0) are represented with blue and grey squares, respectively (left). Percentage of similarity between the model attractors under perturbations (vertical axis) and binarized expression of MEPs and ErPs (horizontal axis) (right). (F) Systematic gene perturbations. The expression states of the model attractor (left). The percentage of similarity between the attractors (vertical axis) and the binarized expressions. (G) The PCA plot of the simulated (perturbed) developmental trajectories are overlaid on the binarized scRNA-seq. The simulated data is color coded by the value of average simulation step (average distance to the attractor of simulation). (PDF) [file pcbi.1009907.s006.pdf]

Fig. S7

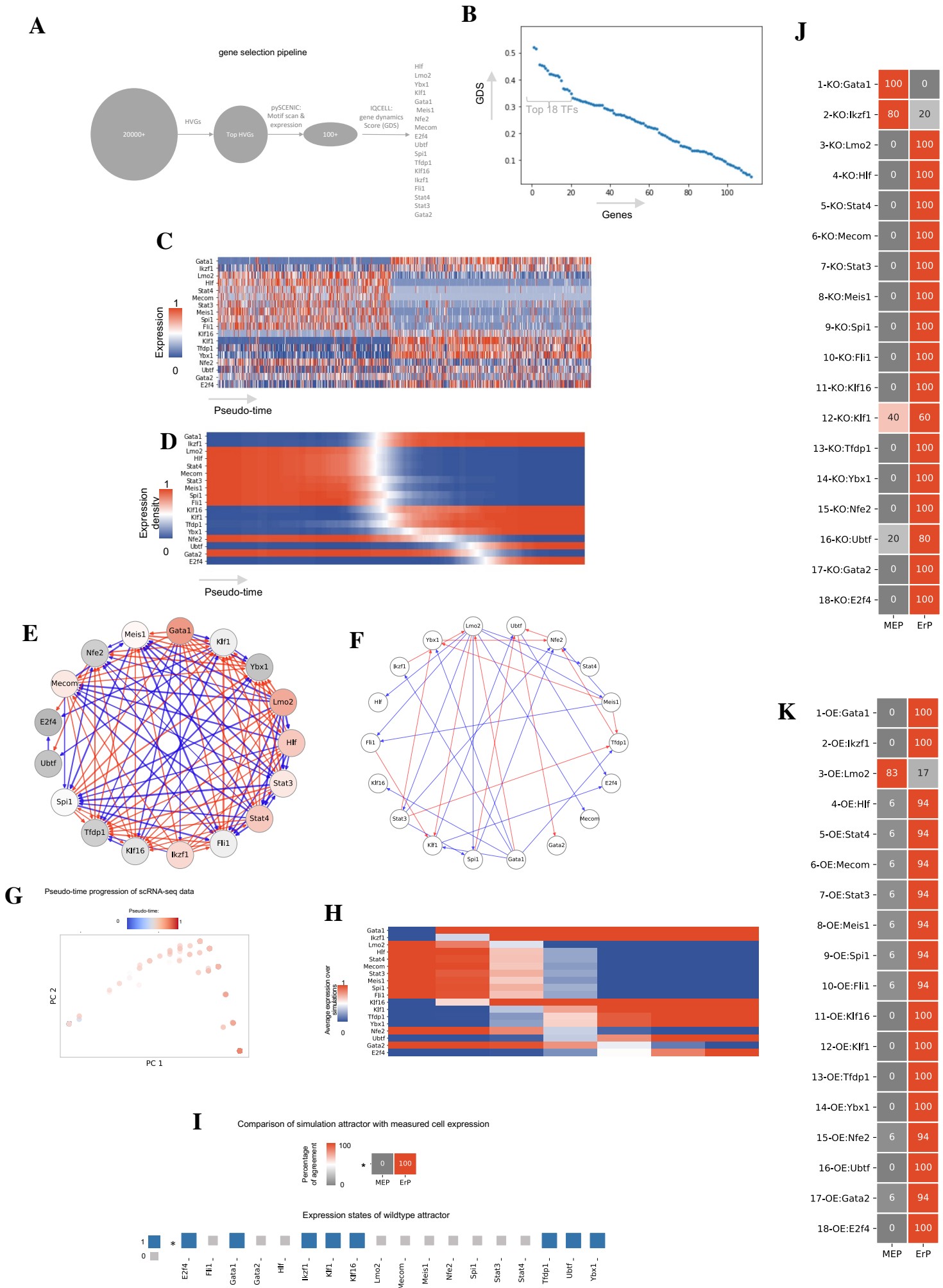

Supplement: S7 Fig — (A) Overview of gene selection procedure for erythropoiesis data. (B) The GDS of genes, the top 14 genes have been selected. (C) Log transformed expression matrix for selected genes from sc-RNA data along the pseudo-time axis. Gene expression is corrected for dropout effects using MAGIC. Red indicates high expression, blue indicates low expression. (D) Smoothed binarized gene expression matrix (expression density). Gene expression values were binarized by clustering, averaged over a pseudo-time window, then sorted based on transition points from early to late. Red indicates high expression, blue indicates low expression. (E) The set of all possible gene-gene interactions, filtered by interaction hierarchy and mutual information. (F) The provisional Boolean GRN for early mouse T-cell development. (G) The PCA plot of the binarized scRNA-seq data color-coded with the pseudo-time values attributed to each cell. The binarization is performed by clustering the scRNA-seq expressions into expressed or not expressed levels. (H) Average gene expression at each simulation step. All simulations started from the same initial condition (MEPs) and move toward the same attractor (*). (I) Expression states of the GRN model steady state attractors. Genes that are expressed (1) and not expressed (0) are represented with blue and grey squares, respectively (top). Percentage of similarity between the model attractors under perturbations (vertical axis) and binarized expression of MEPs and ErPs (horizontal axis) (bottom). (J) Systematic KO: The percentage of similarity between the attractors (vertical axis) and the binarized expressions of MEPs and ErPs (horizontal axis). (K) Systematic OE: The percentage of similarity between the attractors (vertical axis) and the binarized expressions of MEPs and ErPs (horizontal axis). (PDF) [file pcbi.1009907.s007.pdf]

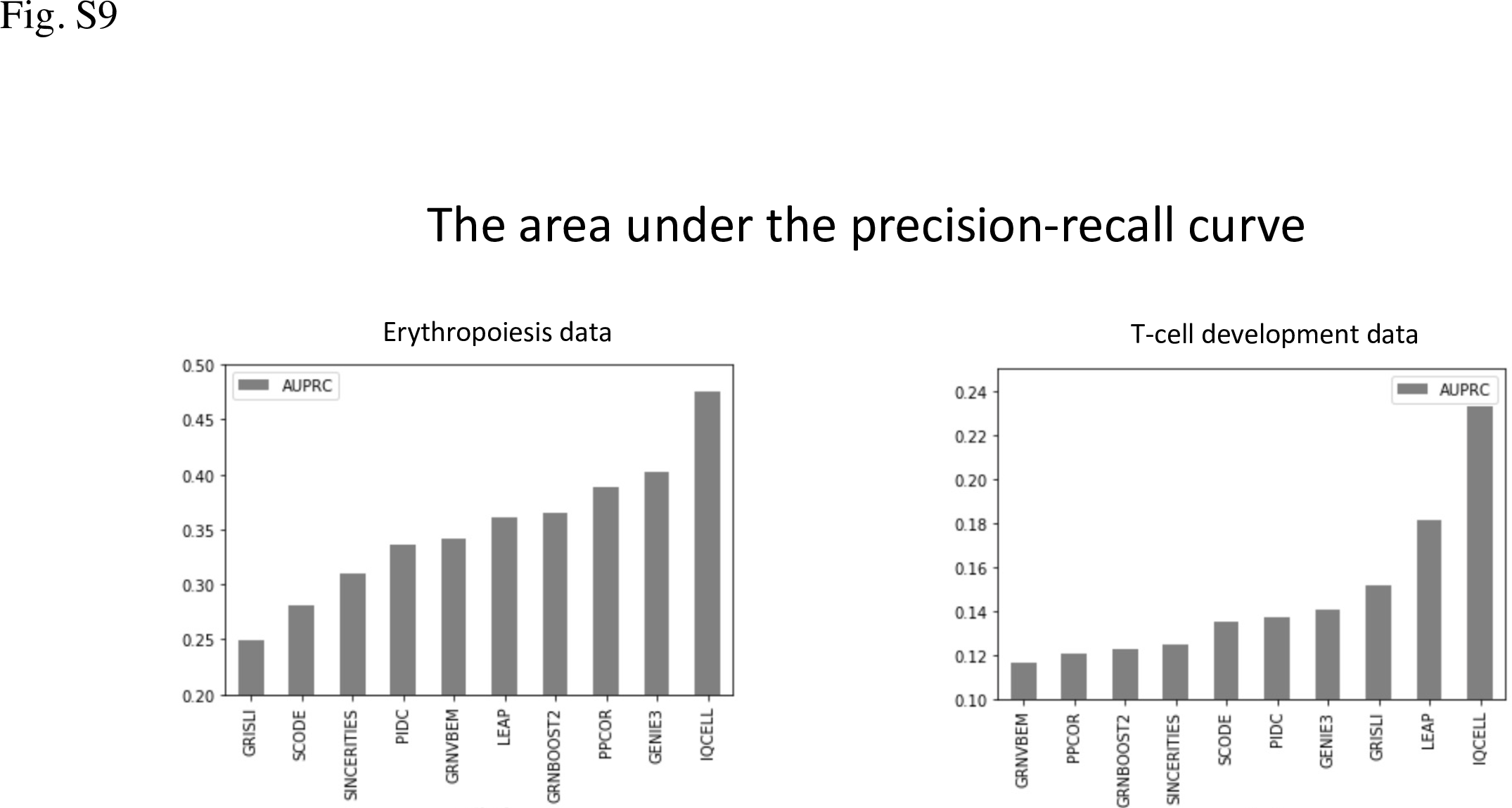

Supplement: S9 Fig — Comparison of the area under the precision-recall curve of IQCELL with other standard GRN inference methods (Pratapa et al., 2020). IQCELL shows improved performance over other methods for both the T-cell and the erythropoiesis datasets. (TIF) [file pcbi.1009907.s009.tif]
