# Supplementary material for: IQCELL: A platform for predicting the effect of gene perturbations on developmental trajectories using single-cell RNA-seq data
Source: PLoS Comput Biol. 2022 Feb 25;18(2):e1009907. doi: 10.1371/journal.pcbi.1009907 (PMC8906617; doi:10.1371/journal.pcbi.1009907)

Fig. S8

A

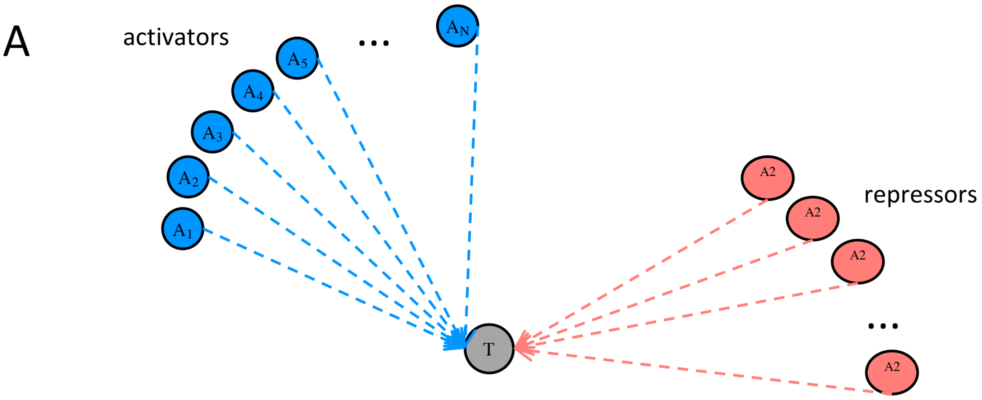

Possible rules for activation $nRules_A = \binom{N}{1} + \binom{N}{2} + \binom{N}{3} + \binom{N}{4}$

Possible rules for repression $nRules_R = \binom{M}{0} + \binom{M}{1} + \binom{M}{2}$

Total possible rules $nRules_T = nRules_A \times nRules_R$

B

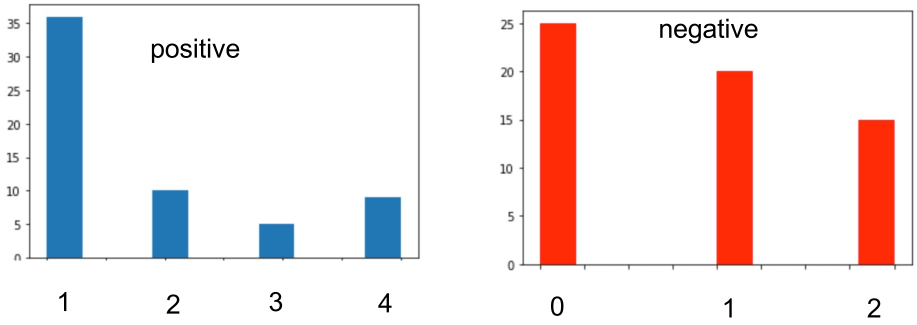

Supplement: S8 Fig — (A) Calculating the number of possible rules for the update function of a gene. (B) Histogram of number of activator/repressors of all genes. (PDF) [file pcbi.1009907.s008.pdf]
