# Supplementary material for: IQCELL: A platform for predicting the effect of gene perturbations on developmental trajectories using single-cell RNA-seq data
Source: PLoS Comput Biol. 2022 Feb 25;18(2):e1009907. doi: 10.1371/journal.pcbi.1009907 (PMC8906617; doi:10.1371/journal.pcbi.1009907)
Supplement: S1 Table — The rules are picked from possible rules in Z3 step based on maximizing the average mutual information per gene. (PDF) [file pcbi.1009907.s010.pdf]

Table S.1

Gene	Update Rule
Bcl11b	((Gata3 and Notch1) and (Runx1 and Tcf7)) and not (Hhex or Spi1)
Cd3e	((Bcl11b and Tcf12) and (Hes1 and Notch1)) and not Lyl1
Ets1	((Bcl11b and Gata3) and (Tcf12 and Runx1)) and not (Hhex or Spi1)
Cd3g	((Bcl11b and Notch1) and (Tcf12 and Hes1)) and not Lyl1
Gata3	((Myb and Tcf7) and Runx1) and not (Hhex or Lmo2)
Hes1	Notch1
Hhex	(Lmo2 and Spi1) and not (Myb and Runx1)
Il7r	((Runx1 and Tcf7) and Myb) and not (Lmo2 and Spi1)
Lef1	(Ets1 and Tcf12) and not Lyl1
Lyl1	(Hhex and Spi1) and not (Bcl11b and Ets1)
Lmo2	Lmo2 and not Notch1
Myb	Tcf7 and not Lmo2
Notch1	Notch1
Ptcra	((Ets1 and Hes1) and (Tcf12 and Notch1)) and not Lyl1
Rag1	((Ets1 and Notch1) and (Tcf12 and Hes1)) and not Lyl1
Runx1	(Myb and Tcf7)
Spi1	Spi1 and not (Runx1 and Tcf7)
Tcf7	Notch1 and not Lmo2
Tcf12	((Notch1 and Runx1) and Bcl11b) and not Hhex
