# Supplementary material for: IQCELL: A platform for predicting the effect of gene perturbations on developmental trajectories using single-cell RNA-seq data
Source: PLoS Comput Biol. 2022 Feb 25;18(2):e1009907. doi: 10.1371/journal.pcbi.1009907 (PMC8906617; doi:10.1371/journal.pcbi.1009907)
Supplement: S2 Table — (PDF) [file pcbi.1009907.s011.pdf]

Table S.2

Gene	
Bcl11a	Itgax
Bcl11b	Kit
Ccr9	Lef1
Cd34	Lmo2
Cd3e	Ly6d
Cd3g	Lyl1
Cd44	Mef2c
Cd82	Meis1
Cebpa	Mpo
Cxcr4	Myc
Dtx1	Mycn
Erg	Nfil3
Ets1	Notch1
Ets2	Nrarp
Flt3	Nt5e
Gata1	Pdgfrb
Gata2	Pgk1
Gata3	Pim1
Gfi1	Ptcra
Gfi1b	Rag1
Hes1	Rag2
Hhex	Runx1
Hoxa9	Runx2
Id2	Runx3
Id3	Sox13
Ikzf1	Spi1
Ikzf2	Spib
Il2ra	Tcf12
Il4ra	Tcf7
Il7r	Tlr7
Irf8	Zbtb16
Itga2b	Zfpm1
Itgam	
