# Supplementary material for: IQCELL: A platform for predicting the effect of gene perturbations on developmental trajectories using single-cell RNA-seq data
Source: PLoS Comput Biol. 2022 Feb 25;18(2):e1009907. doi: 10.1371/journal.pcbi.1009907 (PMC8906617; doi:10.1371/journal.pcbi.1009907)
Supplement: S5 Table — (PDF) [file pcbi.1009907.s014.pdf]

Table S.5

Gene	Update rule
Fli1	Spi1
Gata2	Gata2 and not (Klf1)
Spi1	Spi1 and not (Gata1 and Zfpm1)
Klf1	Gata1 and not Fli1
Gata1	Gata1
Zbtb7a	Klf1
Zfpm1	Gata1
