# Supplementary material for: IQCELL: A platform for predicting the effect of gene perturbations on developmental trajectories using single-cell RNA-seq data
Source: PLoS Comput Biol. 2022 Feb 25;18(2):e1009907. doi: 10.1371/journal.pcbi.1009907 (PMC8906617; doi:10.1371/journal.pcbi.1009907)
Supplement: Attachment — Submitted filename: Response to Reviewers.docx [file pcbi.1009907.s015.docx]

**Reviewer #1:**

Overview:

In this manuscript, Heydari et al. present a new software tool IQCell for inferring and studying gene regulatory networks (GRNs) in scRNA-seq datasets, with a specific focus on applications in stem cells and developmental processes. A key innovation of this tool is that it provides a comprehensive platform for users to infer GRNs directly from scRNA-seq data and then also perform various analyses and simulations. It appears to be assembled from many pre-existing components in a way that is highly useful for the field of scRNA-seq analysis, and includes novel aspects such as segmentation of pseudotime for GRN analysis, a filter for transcription factors to facilitate GRN construction, and GRN model building/testing.

Thanks for your kind words.

The authors demonstrate that their method can effectively recapitulate known GRN structures and behaviors in their test case of T-cell development. This analysis of the T-cell scRNA-seq dataset from (Zhou et al. 2019) is quite extensive, including comparison of GRN-predicted interactions to the experimentally validated gene interactions from (Longabaugh et al., 2017), their own newly-generated scRNA-seq analysis of fetal liver hematopoietic progenitor cells, and comprehensive in silico simulation of gene-level perturbations, performed with their IQCell-derived GRNs. In my opinion, the insight provided by these analyses is largely corroboratory to previous studies, and is used to demonstrate the utility of the IQCell software tool rather than presenting new insight into T cell development in this manuscript. For this reason, I request that the authors and editors consider whether this manuscript would be better served as a Software Submission rather than a Research Article for PLOS Computational Biology. Perhaps this is a minor bookkeeping concern, but I think it’s worth thinking about, because the Software Submission has advantages for this type of paper.

Thank you for this suggestion. Although we agree the paper could fit as either a Software Submission or a Research Article, we have a strong preference to present it as a Research Article. We note that the platform described in this study motivates a design cycle approach to data gathering, simulation, testing / perturbing and ultimately validating. Although we have demonstrated this using two comprehensive datasets, our hope is that IQCELL will motivate new research in a number of dynamic and developmental applications and this fits better as a Research Article.

Along the same lines, if the authors are focused on demonstrating the general utility of the IQCell software tool in this manuscript, then they should test this on more systems rather than just T cell development. I’m generally supportive of this manuscript for publication in PLOS Computational Biology, but if my reading of the authors’ intended focus is correct, then I can’t support publication without validation in additional test systems. My general and specific comments are largely related to this point. If the authors demonstrate that their method can be applied effectively beyond this one well-characterized T cell example application, I will enthusiastically support its publication.

Thank you for your comment. We agree it is important to demonstrate the utility of IQCELL in multiple applications. To this end, we have added a demonstration of IQCELL in Erythrocyte differentiation, and have provided additional benchmarking data. The details of this implementation are provided below.

General Comments:

1. The authors claim that their method, “is generally applicable to scRNA-seq data derived from dynamic developmental systems and should serve as a useful resource for many applications,” and “is not specific to a single developmental system ... may be broadly useful in understanding how GRNs contribute to cell development in a variety of developmental contexts”. However, they have not provided any results from testing their method outside of the context of T-cell development. Can you demonstrate that this pipeline was not essentially overfitted to the specific T-cell developmental context? I assume the ideal utility of this method would be in applying it to under-studied applications to discover GRNs de novo… could this actually be applied where we have not yet mapped out GRNs to assist in manually checking and curating the method outcomes, or where GRNs have different and potentially more complex structures? Analysis of one additional system in addition to T cell development should be sufficient, although more would be very welcome.

Thank you very much for your comment. We had initially chosen the T-cell system because it provided one of the best-studied GRNs in mammalian development. We agree with the necessity of demonstrating IQCELL's performance in another system. Now we have applied IQCELL to Erythropoiesis, and have demonstrated strong performance in the de novo reconstruction of the core GRN in this system. We have tested IQCELL both in the case of curated gene list (with known interactions), where it recovers around 70% of interactions, and also in the case of algorithmic (automated) gene selection, in which it recovered over 70% of the TFs present in the curated gene list. We have also performed a perturbation comparison between the resulting GRNs of curated vs. algorithmically selected genes and showed an 80% match between the two approaches. This excitingly demonstrate the utility of IQCELL going from primary scRNA seq data to executable GRN in two systems. The added results sections are:

**(P.18L.401-P.22L.483) Testing IQCELL performance in Erythropoiesis**

and:

**(P.22L.485-P.22L.497) Testing IQCELL performance via automated TF selection in Erythropoiesis**

2. How frequently can you find scRNAseq datasets that meet the specified quality guidelines? Was this tested on other datasets beyond the two presented in this manuscript to establish these quality criteria? This again gets at how widely applicable this method can actually be.

Thanks for bringing up this point, which is important for the potential users of IQCELL. To address this comment we have added the section below to METHODS, which details two important criteria in data set selection/generation: capturing transitioning cell populations, and sufficient depth of count per cell. More broadly, we believe that with increasing access to scRNAseq data, and with the publication of IQCELL, the availability of this approach as a standard for system GRN simulation will only increase.

**(P.26L.484-P.27L.592) Selecting a suitable scRNA-seq dataset as IQCELL’s input**

There is a direct relationship between the quality of the scRNA-seq data (and the inferred pseudo-time) with the quality of the inferred GRN. A suitable dataset for IQCELL captures the cells in transient states (I.e., along developmental trajectories or other interesting biological transitions) so IQCELL can correctly infer the dynamics of the fate changes. For example, some in-vitro differentiation processes can take several days to happen, and having samples from intermediate days will potentially capture the transient cells. Also, more depth in sequencing will give more confidence in counting TFs, and potentially better performance of IQCELL as the accuracy of calculating mutual information and update rules will increase.

Specific Comments:

1. Please discuss whether the algorithmic decisions, such as using pySCENIC and MAGIC, etc. were based on testing outcomes against alternatives methods? And were these tested with multiple datasets?

Thanks for the comment. MAGIC was selected based on in depth benchmarking papers comparing it to different algorithms. SCENIC was selected because of its unique feature of simultaneous motif and gene expression enrichment. We have added these in the METHODS section:

**(P.27L.600-P.27L.601)** The MAGIC algorithm was selected based on a recent benchmarking paper (Hou et al., 2020) as it outperforms other methods

And:

**(P.28L.635-P.29L.637)** The pySCENIC platform was chosen as it is the only platform (that are aware of) that effectively combines binding motifs and expression enrichment.

2. It would be helpful to provide succinct guidance for users selecting hyperparameters (e.g. k-means clustering or mean-based cutoff for gene expression binarization), perhaps included with the python module documentation.

Thanks for the comment, we have updated the Python module documentation to include the requested guidance. The default values are those we chose in the paper, but we provide the guidance and options of other hyperparameters.

**Reviewer #2:**

The manuscript presents an interesting computational pipeline to reconstruct and simulate Boolean gene regulatory networks from scRNA-seq data. The model requires count matrix, pseudotime ordering and a curated list of genes (which is optional) and allows to simulate temporal dynamics as well as exploring the effect of perturbations, such as knock outs and over expressions. The main weakness of the method is that it has been only applied to one experimental system (T-cell development), therefore making it difficult to prove its generality. I have listed below more specific comments about the model’s formulation and application to the T-cell dataset.

Thanks for your comments. We have now applied IQCELL to another system.

Questions on Model:

Fig. S1B does not really explain how the MAGIC method deals with dropout genes. While I understand that this an already existing method, the main steps of MAGIC should be discussed. Since it is an existing method, adding as supplementary could suffice.

Thanks for the suggestion. We have added a summary of how MAGIC works in the METHODS (in gene expression recovery) :

(P.27L.602-P.27.L605) In Summary, MAGIC calculates the affinities between neighboring cells in high dimensional data and uses this information to recover the undercounted values of gene expression of individual genes (dropouts) from the neighboring cells. This process leads to the imputation of gene expression (van Dijk et al., 2018).

The criteria for gene selection are not very clear, it is only stated that biological information and variation along pseudotime were considered. Was the list curated based on literature, and then refined using the gene dynamics score, etc..?

Thanks for the comment, we apologize for the confusion in the text, we have updated our descriptions. For the T-cell development system, we limited ourselves to the genes that are reported to be important as regulators of ETP, DN2A, and DN2B and DN3 stages, and are in the list of highly variable genes (HVGs). To clarify, we have made the changes below:

(P.8L.182-P.8.L183) After expression recovery, selecting a curated list of genes that are known to be important during early T-cell development (**Table S1** and **Figure 2B**, see METHODs for more detail)

And in METHOD under ‘Selecting a curated gene list for early T-cell differentiation’:

(P.27L.610-P.27.L612) We have curated a list of genes based on literature (Kueh & Rothenberg, 2012; Longabaugh et al., 2017; Ng et al., 2018), and limited ourselves to genes that are included in to HVGs

It would be useful to visualize this gene dynamics score for some selected genes, and examples of genes that did not make the cut based on this variation principle.

Thanks for the great suggestion, we have updated **Figure S.5.A** to include the GDS scores (for all TFs) and highlighted the selected genes.

How do the final networks scale as the threshold to keep the GRN connections is changed?

Thanks for the great comment: To address this, we have updated **Figure S.1.C. The updated figure, shows the selected threshold of keeping the connection (in the initial interaction network), the histogram of distribution of correlation values and also highlights the interactions that will be selected in the final model. Also we have added the below explanation to the METHODs section. In summary, the purpose of the threshold is not to include/exclude reliably signed interactions, but just to make sure that we can assign sign scores (+/-) to the interactions by the means of the sign of their correlation**.

(P.30L.679-P.31L.686) Finally, IQCELL eliminates interactions that are difficult to assign a sing (+/-) to them (their absolute correlation values are smaller than a threshold). The default threshold is permissive so that many interactions can pass and will be signed (for example see **Figure S.1.C**). If an update rule is found for a particular gene, lowering the threshold will only increase the search-time, because it is likely that the link will be selected (due to higher correlation). This threshold allows us to assign a sign (positive or negative) to interactions, its initial purpose is not to cut interactions, but to eliminate interactions with sign ambiguity.

In the methods, it is mentioned that users can optionally include self-regulation, but no more information is provided. However, treating self-regulation seems a fundamentally different problem because IQCELL uses gene-gene correlations to infer the network connections. Please explain.

Thanks for the comment. IQCELL by default doesn’t include self-activation for all genes except in the case of the genes that are active at the beginning of the pseudo-time and don’t have more than 1 active positive input. In the case that self-activation is included, IQCELL counts its correlation as 0 in the final model selection since (as the reviewer pointed out) we cannot use the mutual information method here.

In light of this comment, we more details on this aspect in the METHODS section :

(P.34L.760-P.34L.767) Inclusion of self-activation in the final Boolean GRN model selection will be costly for the update rule since we have set the mutual information of self-activation interaction to ‘0’ which reduces the average mutual information weight of terms that have self-activation. This decision is made because self-activation cannot be weighted effectively by mutual information value against other genes (mutual information is the final criteria used in IQCELL to select the final GRN). However, including self-activation in a Boolean update rule can help the rule to make the cutoff of Z3 step. This ensures that self-activation is only included if it is deemed necessary.

Also, IQCELL discretizes genes expression between Low and High during GRN inference. It would be interesting to see how well some of these genes naturally separate between two ON and OFF states. This is a central assumption in building the Boolean network, since more continuous distributions of gene expression cannot be captured.

Thanks for the comment, to address your point, we have added a new figure that shows the gene expression levels of example genes (Figure S.4.F). As is clear, some genes are separated better than others. We agree with the reviewer and believe that this is a fundamental limitation of binary modeling, which might be resolved by multilevel modeling in future. For more explanation, we have also the below in the discussion.

(P.25L.555-P.25L.561) Although the IQCELL framework allowed us to effectively model regulatory modules as logical (Boolean) gates where no extra parameters are required, logical models are limited in some respects. Firstly, logical modeling cannot effectively capture dose-dependency in gene interactions; for example, it is known that the downstream responses to Gata3 are dose-dependent (Rothenberg, 2019). We suggest in future that this aspect be captured by multilevel models (Collombet et al., 2017). Multilevel modeling requires more sensitive measurements of TF expression levels, which may become feasible with emerging TF profiling methods (Moffitt et al., 2016)

What is the rationale for allowing up to 4 activators and up to 2 inhibitors in the GRN? How does the predicted network change when these numbers are varied (perhaps include some examples as supplementary figures)?

Thanks for the great comment and suggestion. The main limitation, is computational cost. Our choice is similar to other studies that use the Z3 engines for rule construction (Dunn, S. J., et al. 2014; Yordanov, Boyan, et al. 2016; Hamey, Fiona et al. 2017). We have added **Figure S.8** which shows the distribution of edge degree for all genes of the final Boolean GRN in this study. We have added this to the METHODs:

(P.33L.748-P.34L.758) The maximum number of 6 regulators in total is selected due to the computational cost of searching the state-space of possible interactions (**Figure S.8.A**). In summary, the process of finding the rules is costly, since for each possible rule, we calculate the agreement score for thousands of cells along the pseudo-time. Having up to 4 activators and up to 2 repressors, gives rise to up to nRules, where this is defined by (**Figure S.8.A**):

$nRules=[\binom{N}{1}+\binom{N}{2}+\binom{N}{3}$+ $\binom{N}{4}]\times[\binom{M}{0}+\binom{M}{1}+\binom{M}{2}]$

Where N and M are the numbers of all possible activators/repressors in the initial interaction network respectively. Increasing the possible value of activators/repressors will increase ${nRules}_{T}$ and computational cost. As a reference, we have included the distribution of edge degree for all genes of the final Boolean GRN in this study (**Figure S.8.B**).

Also, assuming that all activators must be ON to achieve activation seems a very strict assumption, especially in situations where TFs compete over the same binding sites, and thus not all of them are necessary to start transcription. Is there any evidence for this assumption for the gene lists curated in the manuscript? This could be a case where users should have the possibility to modify these assumptions. Also, it could be interesting to see if relaxing these requirements would fundamentally change the topology of the inferred networks.

Thanks for the great suggestion. We agree with the reviewer that this assumption could be relaxed in some cases. Therefore we have added the capacity of including OR in activation and AND in repression by the user in the platform, although we have kept the original configuration intact for demonstration in this paper. Our suggestion for the user is to first use the original (strict) rules, and if for a particular gene, satisfying rules cannot be found, then proceed to relax the assumption. To keep the package and the manuscript consistent, we have added this sentence to the METHODS:

(P.33L.744-P.33L.747) The assumption of having ‘and’ between activators and ‘or’ between repressors is a strict assumption. IQCELL has the option to relax this assumption to include both ‘and’ and ‘or’ in all rules with the cost of more computational time. We suggest, users first use the default configurations, and in case not find any satisfying rule, then try the relaxed assumptions.

Analysis of T-cell:

It is quite difficult to catch the difference between Fig. 2D and 3A, I suggest some alternative way to plot to highlight the differences. A color shading to visualize time progression would help to characterize the model’s transition states in all PC1-PC2 scatter plots.

We thank the reviewer for the comment, we agree and think that the problem was because of the similar color-coding, we decolored the node for the GRNs in the manuscript and hope it makes the two GRNs look different (in Figures 3 and 6). We also agree that a color shading to visualize time progression would help to characterize the model’s transition states in all PC1-PC2 scatter plots

To address the reviewer’s comment we had to first calculate the average simulation step since the calculation of the average simulation step was not trivial in asynchronous GRNs, so we have added a section in the METHODS to SP to explain what we have done:

(P.35L.791-P.36L.799) To calculate the average step of each simulation data point (e.g. **Figure 3F**), we first find the attractor of interest ( the attractor marked by *), then for each simulation data point (p), we find where that particular point is ($X_{T}^{P}$) for each possible trajectory, then we normalize this value to the length of the trajectory ($L_{T}$):

$$x_{T}^{P}=X_{T}^{P}/L_{T}$$

Where $x_{T}^{P}$ is the scaled place of the point P in trajectory T. Finally for each point, we average over all trajectories:

$$\mathrm{asp}^{P}=<x_{T}^{P}>_{T}$$

which give us the average simulation step ($\mathrm{asp}^{P}$) for each point.

The fact that automatic selection can identify many of the curated genes is a very strong finding. However, it does not justify claiming that automatic feature selection can robustly replace curated lists, as the authors had to include some genes to reproduce their results previously obtained with curated lists.

Thanks for the kind words. Our pipeline only selects TFs. In the case of T cell development, we added Notch1 as an external signal and Rag1 and cd3g stage markers, without those, some biological information about the difference between D2B and DN3 will be lost, and made the comparison impossible. in the case of Erythropoiesis, we didn’t add any markers or signals to the system.

To address this issue across the manuscript, we have replaced the gene selection pipeline with a more accurate term ‘TF selection pipeline’ to emphasize that the pipeline selects TFs and not all genes (stage markers, etc). For example, we have:

(P.16L.349) An automated transcription factor selection module can replace the need for a curated gene list
