# Supplementary material for: IQCELL: A platform for predicting the effect of gene perturbations on developmental trajectories using single-cell RNA-seq data
Source: PLoS Comput Biol. 2022 Feb 25;18(2):e1009907. doi: 10.1371/journal.pcbi.1009907 (PMC8906617; doi:10.1371/journal.pcbi.1009907)
Supplement: Attachment — Submitted filename: respond letter.docx [file pcbi.1009907.s016.docx]

Reviewer #2:

The authors have largely addressed my comments. The manuscript reads well and I am generally in favor of acceptance. My only further suggestion is to test the method on some in silico GRNs in order to compare the results with other existing methods for GRN inference. There are many ODE or boolean models of transcription factor networks that could be used to generate in silico data. One (relatively) quick way to compare to many existing methods is Beeline (<https://www.nature.com/articles/s41592-019-0690-6>). I think this will make the paper much more interesting to people that could actually use the method.

We thank the reviewer for the great suggestion. We have performed the suggested benchmarking analysis and added the results to our manuscript. We are pleased to report that IQCELL outperformed 9 other commonly used platforms, using both the T-cell and Erythropoiesis data sets. The manuscript text has be modified as followed to add these results.

(P:16-L:364) Finally, we compared IQCELL’s performance with other state of the art GRN inference methods (Pratapa et al., 2020) using both the T-cell and Erythropoiesis datasets. Our results show that IQCELL outperformed the other methods, with a higher area under the precision-recall curve (AUPRC) for both datasets (**Figure S9**).

(P:39-L:873) **Calculating area under the precision-recall curve (AUPRC) for IQCELL outputs**

To calculate the area under the AUPRC, we followed Pratapa et al., 2020. For each dataset, we first weighted the gene interactions based on the average mutual information of the rule that the interaction is located in. For duplicated interactions that are present in two or more rules, we picked the highest rank. Next, we weighted the interactions based on their position in the raking and used the Beeline (Pratapa et al., 2020) software to calculate AUPRC for IQCELL and the other methods (**Figure S9**).

(P:47-L:1043) **Supplementary Figure S9.**

Comparison of the area under the precision-recall curve of IQCELL with other standard GRN inference methods (Pratapa et al., 2020). IQCELL shows improved performance over other methods for both the T-cell and the erythropoiesis datasets.

Reviewer #3: The authors have adequately addressed the questions raised by the reviewer #1.
 In the current manuscript, the pseudo-time ordering is inferred by DDRtree, which is not the popular one among many pseudo-time inference methods. I wonder whether other pseudo-time methods would affect the result?

We thank the reviewer for the question. In our T-cell dataset analysis, we used the DDRtree method for reconstructing the developmental trajectory. This was in line with the analysis of the original paper that the dataset is adapted from. In our Erythropoiesis data analysis, we have used the Slingshot platform (as a commonly used method for trajectory inference) for reconstructing the developmental trajectory. We have added these details to our methods section:

(P:39-L:872) We processed the erythroid differentiation dataset the same way as in the original publication (Nestorowa et al., 2016) to better support our comparisons; specifically we used the Slingshot method to infer the developmental trajectory (Street et al., 2018).

Also, the applications seem only for single trajectory. How to apply the tool to the branching trajectories should be considered or discussed at least.

Thank you for your comment, we have added a sentence to our discussion to provide insight into how to handle branching trajectories:

(P:20-L:1434) Here we have tested IQCELL’s performance in single developmental trajectories. However, IQCELL could be used to infer GRNs for multiple developmental trajectories (e.g. trajectories with bifurcations) by inferring candidate GRNs for each trajectory.

In addition, I have some suggestions as follows:

1. Now in the manuscript the network edges were all visualized as arrows for both positive and negative regulations. However, it’s better to distinguish them using shear head arrows and round head arrow, as most GRNs commonly appear.

Thank you for your suggestion, we have updated the provisional GRNs to the requested format (Figures 3A and 6C).

2. Although the figures are in 300 dpi resolution, but some fonts in the figures are not very clear. The authors may need to improve them.

We have updated the font sizes in all of the Figures (1,2,3,4,5,6) to make sure the text is readable.

3. The manuscript should be carefully proofread and edited. Some typos (e.g.,): "Transcription factor" should be consistently "transcription factor"; Line 140.

Thank you for bringing this to our sight. We have carefully proofread and edited the manuscript.
